# Olecranon fossa osteoid osteoma excision through arthroscopy: surgical technique

**DOI:** 10.1093/jscr/rjad720

**Published:** 2024-01-16

**Authors:** Kushtrim Grezda, Arber Lama, Dren Kusari, Qendrim Hajdari

**Affiliations:** Department of Orthopedics and Traumatology, University Clinical Centre of Kosova, Medical Faculty, University of Prishtina, 10000 Prishtina, Kosovo; University Hospital of Basel, 4031 Basel, Switzerland; Department of Orthopedics and Traumatology, University Clinical Centre of Kosova, Medical Faculty, University of Prishtina, 10000 Prishtina, Kosovo; Evidence Synthesis Group, 10000 Prishtina, Kosovo; Department of Orthopedics and Traumatology, University Clinical Centre of Kosova, Medical Faculty, University of Prishtina, 10000 Prishtina, Kosovo; Department of Orthopedics and Traumatology, University Clinical Centre of Kosova, Medical Faculty, University of Prishtina, 10000 Prishtina, Kosovo

**Keywords:** osteoid osteoma, olecranon fossa, arthroscopy

## Abstract

In the elbow joint, occurrences of intra-articular osteoid osteoma are uncommon. We detail the case of a 21-year-old male who experienced pain, inflammation, and a restricted ability to move his elbow. For a few months, the diagnoses was missed and the patient was treated for idiopathic synovitis. After a contrast MRI, the tumor was revealed. During an arthroscopic examination of the elbow, a distinct red lesion was observed after the removal of the pale reactive bone in the olecranon cavity. This was subsequently removed in its entirety with the aid of a specialized bone tool. Histopathology confirmed the diagnosis of osteoid osteoma. Remarkably, the individual reported alleviation from the symptoms just a day following the operation and regained full range of motion 5 weeks after the surgery. This case underscores the efficacy of arthroscopy in addressing intra-articular osteoid osteoma, with a focus on accurately pinpointing the lesion.

## Introduction

Osteoid osteoma (OO) is a solitary benign osteoblastic bone tumor which first was described by Bergstrand in 1930 and later characterized by Jaffe in 1935 [1]. It is third most common benign bone tumor with incidence of 11% [2]. This tumor appears more often in individuals between ages of 5 and 25 years old with a 3–1 male preponderance [3]. Usually OO are small in size between <1.5 and 2 cm and they appear within cortex of the long bones of lower limbs [4]. Typical clinical presentations of OO are pain that it worsens at night and it is improved by NSAIDS and use of aspirin, but when the OO is inside the joint, it is accompanied with synovitis, limited range of motion, joint effusion, and contractures [5].

Intra-articular elbow OO diagnosis is very challenging because localization of this tumor in elbow joint is very rare [6], and other confounding factors such as previous supracondylar fracture or other differential diagnosis can lead to a delayed diagnosis or even misdiagnosis [7].

Open excision of the OO has been reported in the literature as a successful procedure [8]. On the contrary, arthroscopic removal may represent a more favorable alternative, as it is a minimally invasive procedure and may be deemed more appropriate from a cosmetic perspective.

## Case report

**Figure 1:**
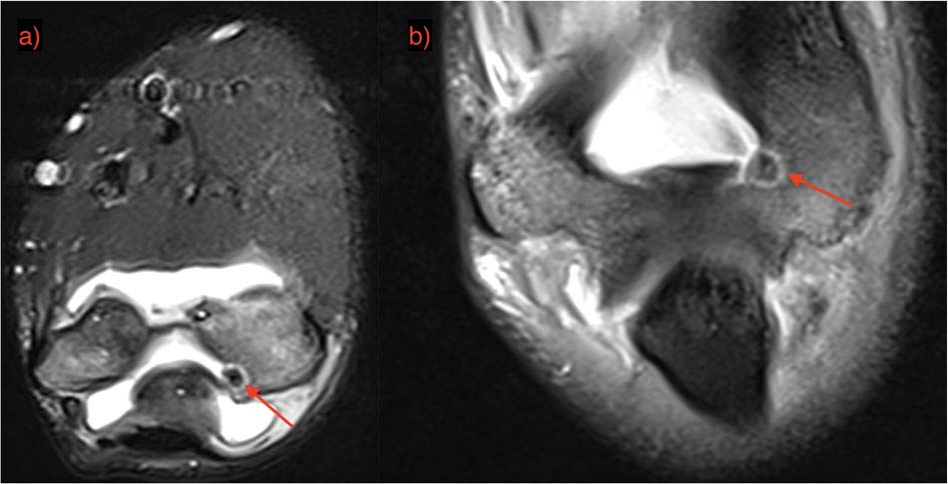
OO localization with contrast MRI: (a) coronal view and (b) frontal view.

**Figure 2:**
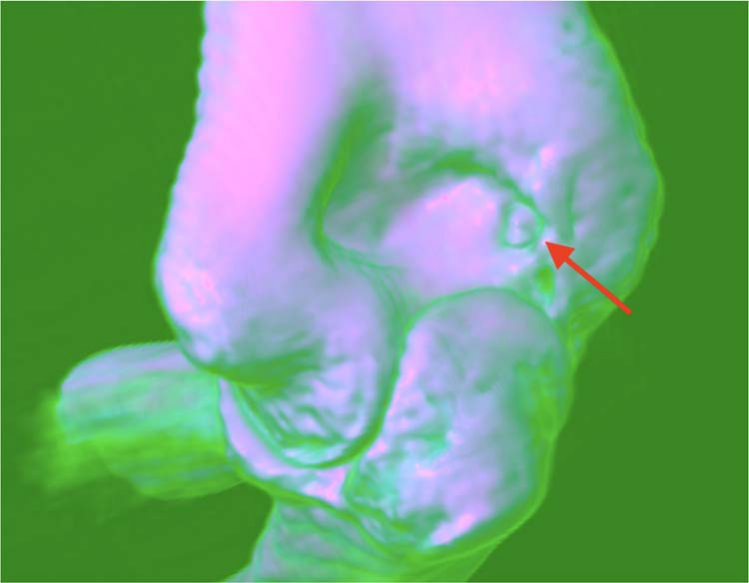
OO localization with 3D CT scan.

A 21-year-old man, who is right-hand-dominant, presented in our clinic with a localized and persistent pain in his right elbow joint that had been present for more than 6 months. Previously, the patient was treated by Rheumatologist for several months with initial diagnosis as idiopathic synovitis involving the right elbow and he was referred to us for a second opinion. He reported that the complains started when he was lifting weights in the gym. On physical examination, a swelling was noted and a restricted extension/flexion (−45°/115°) was present, while pronation/supination was within normal values. There was pain in terminal extension but also during the night. The neurovascular examination was normal. Laboratory tests and plain radiography results were normal. Subsequent MRI with contrast was performed after normal plain radiography results, and it revealed a focal nidus in olecranon fossa (Figs 1 and 2) consistent with an intra-articular OO. Then, a 3D CT scan was also performed for better localization of the tumor [3].

The surgery was performed with the patient under general anesthesia and lateral decubitus position. A pressurized cuff was wrapped around the arm. Before the introduction of the 4.0-mm arthroscope, the elbow cavity was filled with 20-mL saline solution. Through the central posterior portal an abnormal growth manifested as a raised, blood-engorged bony bump accompanied by inflammation nearby was encountered. The core of the OO and surrounding hardened bone was excised. The removed tissue underwent a detailed lab analysis. Any leftover inflamed bone structures were thoroughly removed using an electrically powered surgical drill. The final lab results verified the presence of an OO.

Following the surgery, there were not any complications. Three days after the procedure, the patient indicated a decrease in discomfort. Two weeks after the surgery, the patient returned to work. By the 5th-week check-up, full ROM was encountered.

## Discussion

OO is a prevalent benign bone with typical radiographic finding as radiolucent center, often encircled by reactive bone thickening. While frequently found in the shafts of long bones, its presence within joints, such as the elbow, is extraordinarily unusual. Various studies mention its occurrence in areas such as the head, upper ulna, ulna’s coronoid process, capitellum, and lower humerus [9].

Prolonged intrajoint lesions might lead to conditions such as synovitis, cartilage wear, and joint stiffness. These intrajoint occurrences can be mistaken for conditions such as arthritis or tenosynovitis. Because of its mimicking nature, the diagnosis of elbow OO can be challenging. Conventional X-rays often miss the radiolucent center due to minimal reactive thickening around it. CT scans with detailed sectioning can offer better diagnostic precision, especially when MRI’s effectiveness is still debated upon [10]. Notably, a distinct response to anti-inflammatory drugs such as aspirin is typical of OO. However, for intrajoint lesions, these medications seem less effective. Pain relief can be sought through NSAIDs. Traditional treatment approaches often involve a surgical removal of the growth. But, minimally invasive procedures such as radiofrequency ablation or CT-guided removal are becoming more popular, especially for hard-to-access lesions [11]. Elbow arthroscopy, in particular, is on the rise, offering several advantages such as reduced infection risks, minimal scarring, and quicker postoperative recovery [12].

Utilizing arthroscopic ablation, a study was conducted on 10 patients [13]. The findings from this study revealed a significant challenge; a majority of these patients could not undergo a histological diagnosis. The primary reason for this diagnostic challenge was attributed to the limitations of the arthroscopic method, which often provided insufficient samples for a thorough examination. In a separate report by Zupanc and his colleagues, they detailed an instance involving a 42-year-old male patient [14]. This patient was experiencing symptoms indicative of a juxta-articular OO, specifically located at the capitellum. To address this, an arthroscopic excision was undertaken. However, during the procedure, the surgical team used a motorized shaver to remove the nidus of the condition. This approach inadvertently fragmented the lesion. As a result of this fragmentation, the obtained samples were deemed unsuitable for a conclusive histopathological diagnosis.

Though some studies detail an arthroscopic removal of OOs in elbows, we opted for this method, granting a comprehensive view for precise lesion targeting [13–15]. After lesion removal, any existing flexion contractures typically resolve on their own. But in cases of severe motion restrictions, a capsular release might be necessary. In our presented case, the elbow’s movement range was unhampered, making capsular release unnecessary.

## Conflict of interest statement

No conflict of interest is declared by the authors.

## Funding

None declared.

## Informed consent

Patient consent was obtained before writing the article.

## Supplementary Material

Submission_Video_rjad720
